# Association of early bedtime at 3 years of age with higher academic performance and better non-cognitive skills in elementary school

**DOI:** 10.1038/s41598-023-48280-5

**Published:** 2023-11-27

**Authors:** Masahiro Nishiyama, Yuki Kyono, Hiroshi Yamaguchi, Aoi Kawamura, Shizuka Oikawa, Shoichi Tokumoto, Kazumi Tomioka, Kandai Nozu, Hiroaki Nagase

**Affiliations:** 1 Department of Pediatrics, Kobe University Graduate School of Medicine, 7-5-2 Kusunoki-cho, Chuo-ku, Kobe, Hyogo 650-0017 Japan; 2 Department of Neurology, Hyogo Prefectural Kobe Children’s Hospital, Kobe, Hyogo Japan

**Keywords:** Paediatric research, Paediatric research, Epidemiology, Intelligence

## Abstract

This study investigated the relationship between sleep habits in early childhood and academic performance and non-cognitive skills in the first grade. We retrospectively analyzed a longitudinal population-based cohort from birth through early childhood, up to elementary school, in Amagasaki City, Japan. The primary outcome was academic performance in the first grade. Other outcomes were self-reported non-cognitive skills. Overall, 4395 children were enrolled. Mean national language scores for children with bedtimes at 18:00–20:00, 21:00, 22:00, and ≥ 23:00 were 71.2 ± 19.7, 69.3 ± 19.4, 68.3 ± 20.1, and 62.5 ± 21.3, respectively. Multiple regression analysis identified bedtime at 3 years as a significant factor associated with academic performance. However, sleep duration was not significantly associated with academic performance. Bedtime at 3 years also affected non-cognitive skills in the first grade. Diligence decreased with a later bedtime (21:00 vs. 18:00–20:00; odds ratio [OR]: 1.98, 95% confidence interval [CI] 1.27–3.09; 22:00 vs. 18:00–20:00; OR: 2.15, 95% CI 1.37–3.38; ≥ 23:00 vs. 18:00–20:00; OR: 2.33, 95% CI 1.29–4.20). Thus, early bedtime at 3 years may be associated with a higher academic performance and better non-cognitive skills in the first grade. Optimum early-childhood sleep habits may positively impact academic future.

## Introduction

Adequate duration and quality of sleep influences the physical health and learning in children^1–3^. Several studies have demonstrated that sleep is associated with academic performance and cognitive skills^3–5^. Experimental and cross-sectional studies suggest that insufficient and mistimed sleep is associated with reduced cognitive function^4^.

Cognitive function is crucial in social success; specifically, cognitive development and academic achievement in children highly influence social and economic success in adulthood^6,7^. Recently, the role of non-cognitive skills has also been in focus^8^. These non-cognitive skills include executive function, inhibitory control, self-esteem, diligence, and emotional regulation^8^. The Perry Preschool Program, which was an early childhood education program conducted at the Perry Elementary School in Michigan in the early 1960s, revealed the importance of non-cognitive skills as well as cognitive function^9^. The study also emphasized the importance of education in early childhood^9^. In developed countries with declining birth rates, there is a growing interest in the characteristics of early childhood environments that are best for the future of children.

Although an association between sleep habits and academic performance has been well established^4,10^, the ideal sleep habits supporting cognition is inconclusive. Poor sleep quality and insufficient sleep are associated with inferior academic performance^1,4^. Contrarily, another study identified no cross-sectional association between sleep duration and cognition in elementary school-aged children^11^. Moreover, longitudinal cohort studies in early childhood are limited^12,13^. Therefore, the impact of early-childhood sleep habits on academic performance in later years remains unclear. Furthermore, the influence of early childhood sleep habits on non-cognitive skills is unknown.

This study followed a longitudinal population-based cohort from birth, through early childhood up to elementary school. The present study aimed to investigate the relationship between sleep habits in early childhood and academic performance and non-cognitive skills in the first grade of elementary school.

## Participants and methods

### Study design and population

This longitudinal population-based retrospective cohort study utilized records from municipal health check-ups and academic survey in Amagasaki City, Japan. We accessed the deidentified data on health check-ups and academic research after approval by the review board at the Planning and Coordination Bureau of Amagasaki City (dated July 12, 2021; no. 215). The requirement for informed consent was waived because the data were anonymized, which was approved by the review board at the Planning and Coordination Bureau of Amagasaki City. The analyses were carried out at the Kobe University Graduate School of Medicine. All study methods were in accordance with the approved guidelines.

**Figure 1:**
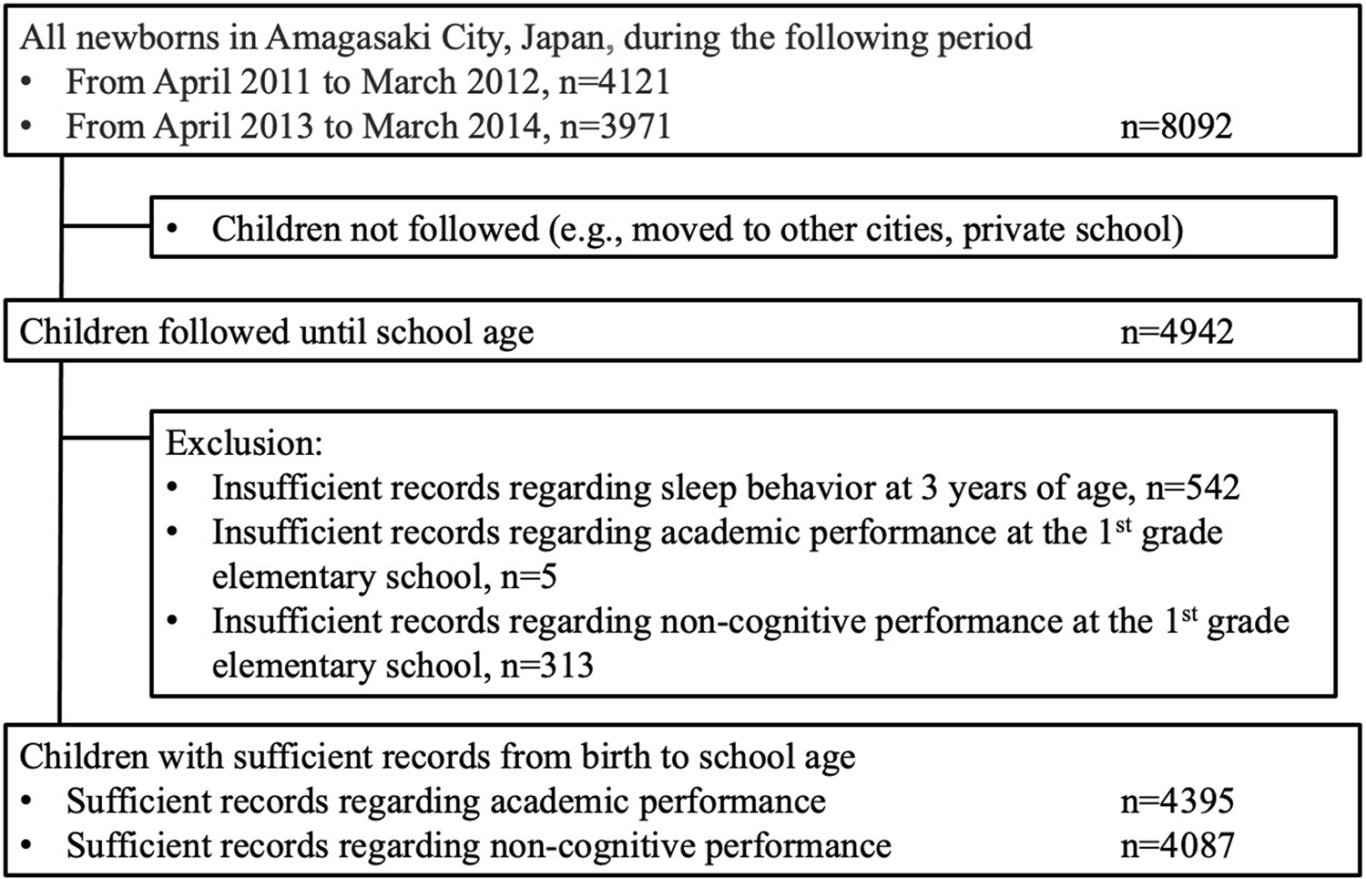
Flow chart of the participants.

Amagasaki City is located in the southern side of the main island of Japan, with a population of approximately 450,000 and an annual birth rate of approximately 4000. All pregnant women and their children in Amagasaki City participated in the health check-up program from pregnancy to 3 years of age. This study included children born in Amagasaki City between April 2011 and March 2012, or April 2013 and March 2014; they were followed until school age (Fig. 1). Furthermore, these children attending first grade at public elementary schools in Amagadaki City took an academic survey at the following timepoints: December 2018 and December 2020, which is the 8th month of elementary school. Children who moved to other cities or enrolled in a private elementary school were not followed. Thereafter, they comprised 4942 children, of whom we excluded 542 children without sufficient records regarding sleep behavior at 3 years of age. Further, five children without sufficient records regarding academic performance were excluded from its analysis and 313 children without sufficient records regarding non-cognitive skills were excluded from its analysis. Finally, 4395 children were analyzed regarding academic performance, and 4087 children were analyzed regarding non-cognitive skills.

### Measurements

Birth data including sex, birth month, gestational age, maternal age at child’s delivery, and maternal smoking at pregnancy confirmation were collected from the maternal and neonatal health records, managed by Amagasaki City Health and Welfare Bureau. Information on lifestyle at 3 years of age were collected using questionnaires and cross-checked by public health nurses when the children visited Amagasaki City Public Health Centers. The questions included bedtime (e.g., 18:00, 19:00, 20:00, 21:00, 22:00, and 23:00), wake-up time, and hours spent watching television or electronic media. The presence or absence of mental developmental delay was assessed by a public health nurse and confirmed by a pediatric physician during the check-up at 3 years of age. The economic status of the caregiver when the child was in first grade at elementary school was categorized as no financial assistance for school attendance, some financial assistance, and full financial assistance. For a typical household of three people, some financial assistance is provided if the annual income is < 2,388,000 yen, and full financial assistance is provided if the annual income is < 1,800,000 yen.

Academic performance was evaluated using a national language (Japanese) and math test in the first grade. The test was originally prepared for this research project at the Amagasaki City Institute for Learning and Growing according to governmental educational guidelines by the Ministry of Education, Culture, Sports, Science and Technology.The test was conducted during school hours and was supervised by a school teacher. A perfect score on the test was 100 points. Assessed non-cognitive skills included self-esteem, diligence, and kindness. Non-cognitive skills were evaluated exclusively using the children’s answers to the following questions: (1) self-esteem—do you think you are a good person?, (2) diligence—do you work hard at everything you do?, and (3) kindness—are you a compassionate person? Children answered with either “yes” or “no” for each question. These questions were also originally prepared for this research project at the Amagasaki City Institute for Learning and Growing.

### Statistical analysis

The primary outcome was academic performance in the national language test in the first grade of elementary school. The secondary outcome was academic performance in math. The other outcomes included non-cognitive skills, namely self-esteem, diligence, and kindness. Predictor variables were bedtime (18:00–20:00, 21:00, 22:00, and ≥ 23:00) and sleep duration 6–8, 9, 10, and ≥ 11 h) at 3 years of age. Covariates included sex, birth month, gestational age, maternal age at delivery, maternal smoking, economic status, television or electronic media viewing at 3 years of age, and mental developmental delay at 3 years of age. Birth month was categorized as April–September or October–March. In Japan, the school year begins in April, and those born in April are around 12 months ahead of those born in March. Maternal age at delivery was categorized as < 20 years, 20–34 years, or ≥ 35 years. Television or electronic media viewing was categorized as < 2, 2–3, and ≥ 4 h.

We aimed to investigate the relationship between sleep habits in early childhood and academic performance and non-cognitive skills in the first grade of elementary school, with particular focus on bedtime. However, because previous studies have mainly targeted sleep duration, we initially performed a rough analysis of both bedtime and sleep duration. First, academic performance was compared according to the variables (bedtime or sleep duration). An additional analysis for variables was performed if there was a significant difference. Characteristics were categorized according to significant variables. Further, we used a linear regression model to analyze the relationship between academic performance and variables. We further performed a multiple regression analysis adjusted for these covariates. Finally, we used multiple logistic regression analysis to investigate the relationship between non-cognitive skills and variables. We also compared characteristics regarding birth data between included (n = 4395) and excluded cases (n = 3697).

Results are expressed as number (%), estimates and standard errors (SE) in the linear regression model and odds ratio (OR) with 95% confidence interval (CI) in the logistic regression analysis. One-way analysis of variance (ANOVA), t-tests with Bonferroni correction, and Fisher’s exact tests were performed as appropriate. A P-value of < 0.05 was considered statistically significant for all tests. Analyses were performed using JMP, version 13.0 (SAS Institute, Cary, NC) and EZR (Saitama Medical Center, Jichi Medical University, Saitama, Japan), which is a graphical user interface for R (version 3.1.2; The R Foundation for Statistical Computing, Vienna, Austria)^14^.

## Results

### Academic performance according to bedtime or sleep duration at 3 years of age

**Figure 2:**
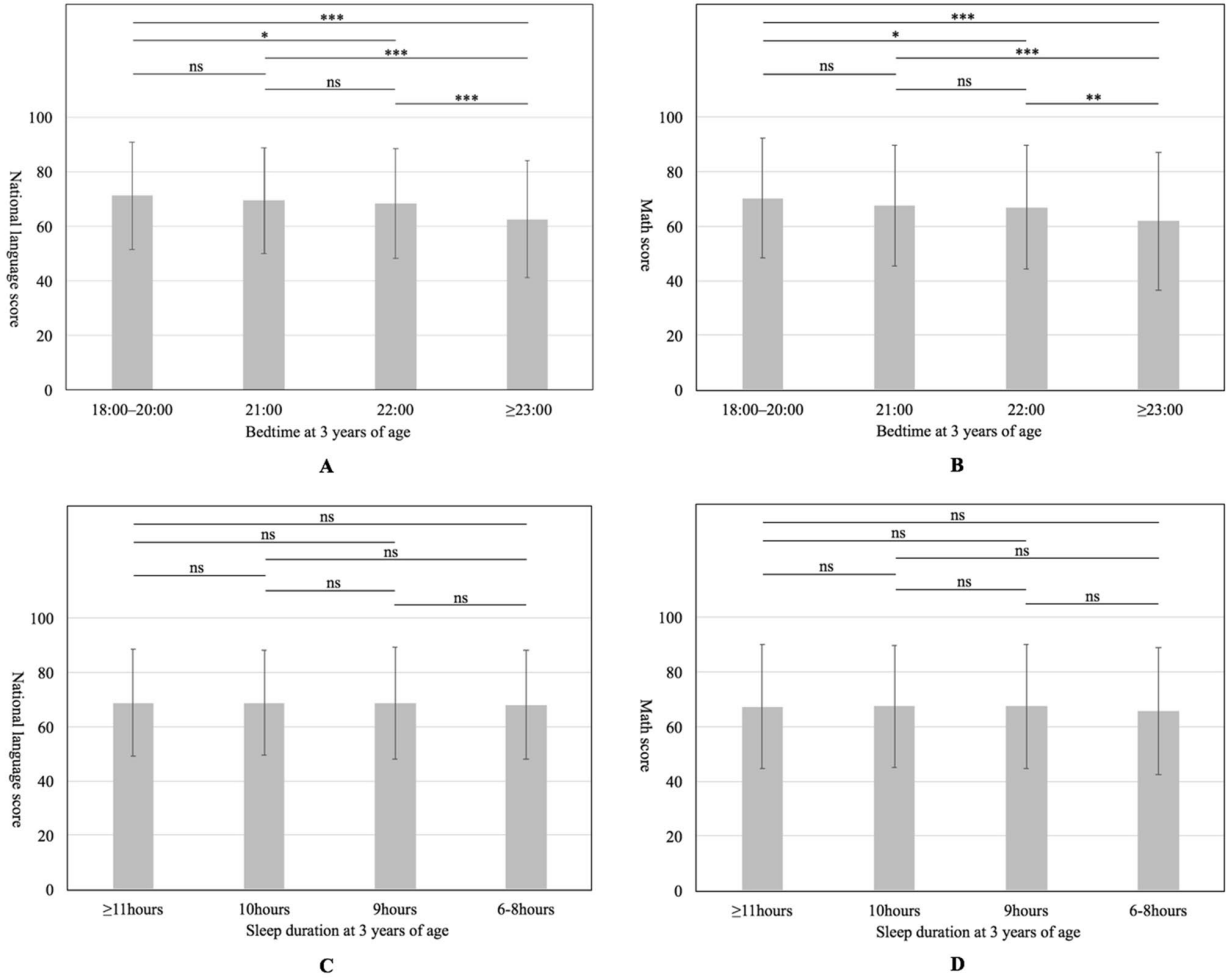
Comparisons of national language and math scores based on the bedtime and sleep duration at 3 years of age. (**a**) Comparison of national language scores based on the bedtime at 3 years of age: 18:00–20:00 (n = 506), 21:00 (n = 2045), 22:00 (n = 1555), ≥ 23:00 (n = 289). (**b**) Comparison of math scores based on the bedtime at 3 years of age; 18:00–20:00 (n = 506), 21:00 (n = 2045), 22:00 (n = 1555), ≥ 23:00 (n = 289). (**c**) Comparison of national language scores based on sleep duration at 3 years of age; ≥ 11 h (n = 850), 10 h (n = 1913), 9 h (n = 1391), 6–8 h (n = 221). (**d**) Comparison of math scores based on the sleep duration at 3 years of age; ≥ 11 h (n = 850), 10 h (n = 1913), 9 h (n = 1391), 6–8 h (n = 221). The boxes shows the mean values. The upper and lower whiskers represent standard deviations. Data were analyzed using the t-test with Bonferroni correction. *p < 0.05, **p < 0.01, ***p < 0.001. ns; not significant.

Figure 2 shows the academic performance according to bedtime or sleep duration. Both national language and math scores differed according to bedtime (one-way ANOVA, *P* < 0.001). Contrarily, there were no significant differences in either national language or math scores according to sleep duration (one-way ANOVA, national language, *P* = 0.961; math, *P* = 0.735). The mean academic performance scores according to sleep duration were as follows: national language: ≥ 11 h, 68.8 ± 19.6; 10 h, 68.8 ± 19.5; 9 h, 68.8 ± 20.6; 6–8 h, and 68.1 ± 19.9; math: ≥ 11 h, 67.3 ± 22.6; 10 h, 67.4 ± 22.2; 9 h, 67.4 ± 22.7; and 6–8 h, 65.6 ± 23.3.

### Characteristics of children according to bedtime at 3 years of age

Table 1 describes the baseline characteristics of the 4395 enrolled children, categorized into four groups according to bedtime. Of these, 506 (11.5%) went to bed at 18:00–20:00, 2045 (46.5%) at 21:00, 1555 (35.4%) at 22:00, and 289 (6.6%) at 23:00 or later. The mean academic performance in national language in the first grade of elementary school was 68.7 ± 19.9 and that in math was 67.2 ± 22.5. Children who went to bed earlier showed higher academic performance (national language: 18:00–20:00, 71.2 ± 19.7; 21:00, 69.3 ± 19.4; 22:00, 68.3 ± 20.1; and ≥ 23:00, 62.5 ± 21.3; math: 18:00–20:00, 70.3 ± 21.8; 21:00, 67.6 ± 22.0; 22:00, 66.9 ± 22.6; and ≥ 23:00, 61.8 ± 25.4).

**Table 1:** Characteristics of 4395 children according to bedtime at 3 years of age.

	Bedtime at 18:00–20:00 (n = 506)	Bedtime at 21:00 (n = 2045)	Bedtime at 22:00 (n = 1555)	Bedtime at ≥ 23:00 (n = 289)	Total (n = 4395)
Academic performance in national language in the first grade of elementary school, mean ± SD	71.2 ± 19.7	69.3 ± 19.4	68.3 ± 20.1	62.5 ± 21.3	68.7 ± 19.9
Academic performance in math in the first grade of elementary school, mean ± SD	70.3 ± 21.8	67.6 ± 22.0	66.9 ± 22.6	61.8 ± 25.4	67.2 ± 22.5
Sex					
Male	275 (54.3%)	1036 (50.7%)	769 (49.5%)	158 (54.7%)	2238 (50.9%)
Female	231 (45.7%)	1036 (50.7%)	786 (50.5%)	131 (45.3%)	2157 (49.1%)
Gestational age (weeks), mean ± SD^a^	38.7 ± 1.6	38.8 ± 1.6	38.8 ± 1.6	38.9 ± 1.7	38.8 ± 1.6
22–32	6 (1.2%)	16 (0.8%)	11 (0.7%)	1 (0.3%)	34 (0.8%)
33–36	21 (4.1%)	81 (4.0%)	65 (4.2%)	18 (6.2%)	185 (4.2%)
37–43	479 (94.7%)	1948 (95.2%)	1478 (95.1%)	270 (93.4%)	4175 (95.0%)
Birth month					
April–September	238 (47.0%)	1074 (52.5%)	791 (50.9%)	161 (55.7%)	2264 (51.5%)
October–March	268 (53.0%)	971 (47.5%)	764 (49.1%)	128 (44.3%)	2131 (48.5%)
Maternal age at delivery (years), mean ± SD^c^	31.4 ± 4.6	31.4 ± 5.1	31.3 ± 5.5	30.4 ± 6.0	31.3 ± 5.2
< 20	3 (0.7%)	21 (1.3%)	22 (1.8%)	14 (6.2%)	60 (1.7%)
20–34	324 (78.6%)	1193 (73.3%)	899 (71.9%)	152 (67.0%)	2568 (73.0%)
≥ 35	85 (20.6%)	413 (25.4%)	329 (26.3%)	61 (26.9%)	888 (25.3%)
Maternal smoking at pregnancy confirmation^d^					
No	361 (10.0%)	1337 (13.7%)	946 (20.5%)	163 (23.5%)	2807 (83.7%)
Yes	40 (90.0%)	212 (86.3%)	244 (79.5%)	50 (76.5%)	546 (16.3%)
Economic status					
No financial assistance for school attendance	437 (86.4%)	1738 (85.0%)	1305 (83.9%)	223 (77.2%)	3703 (84.3%)
Some financial assistance for school attendance	60 (11.9%)	271 (13.2%)	210 (13.5%)	48 (16.6%)	589 (13.4%)
Full financial assistance for school attendance	9 (1.8%)	36 (1.8%)	40 (2.6%)	18 (6.2%)	103 (2.3%)
Television or electronic media viewing at 3 years of age (h)^b^					
< 2	154 (31.3%)	633 (31.7%)	402 (26.5%)	48 (17.0%)	1237 (28.9%)
2–3	265 (53.9%)	1092 (54.8%)	868 (57.2%)	145 (51.2%)	2370 (55.3%)
≥ 4	73 (14.8%)	269 (13.5%)	247 (16.3%)	90 (31.8%)	679 (15.8%)
Mental developmental delay at 3 years of age					
No	490 (96.8%)	1974 (96.5%)	1508 (97.0%)	273 (94.5%)	4245 (96.6%)
Yes	16 (3.2%)	71 (3.5%)	47 (3.0%)	16 (5.5%)	150 (3.4%)

^a^Data missing for < 1% children.

^b^Data missing for 1%–10% children.

^c^Data missing for 10%–20% children.

^d^Data missing for 20%–30% children.

SD, standard deviation.

### Relationships among academic scores and variables at 3 years of age

Tables 2 and 3 show the relationship between academic performance in the first grade of elementary school and bedtime at 3 years of age. Unadjusted analysis identified bedtime at 3 years of age and all covariates to be associated with academic performance in the national language test. The scores were 2.9 points lower for bedtime at 22:00 and 8.65 points lower for bedtime at 23:00 than for bedtime at 18:00–20:00. Multiple regression analysis adjusted for the above covariates revealed that bedtime was associated with academic performance in the national language test.

**Table 2:** Relationship between academic performance in national language in the first grade of elementary school and bedtime at 3 years of age.

		Unadjusted					Adjusted (n = 3268)^a^			
		Estimate	SE	t value	p value		Estimate	SE	t value	p value
						(Intercept)	67.5	2.91	23.24	< 0.001
Bedtime at 3 years of age										
18:00–20:00	(Intercept)	71.18	0.88	80.73	< 0.001		0 (Reference)			
21:00		– 1.86	0.98	– 1.89	0.059		– 2.16	1.07	– 2.02	0.044
22:00		– 2.90	1.01	– 2.85	0.004		– 2.35	1.10	– 2.13	0.034
≥ 23:00		– 8.65	1.46	– 5.92	< 0.001		– 6.30	1.63	– 3.86	< 0.001

^a^Adjusted for sex, gestational age, birth month, maternal age at delivery, maternal smoking at pregnancy confirmation, economic status, television or electronic media viewing at 3 years of age, mental developmental delay at 3 years of age.

SE, standard error.

**Table 3:** Relationship between academic performance in math in the first grade of elementary school and bedtime at 3 years of age.

		Unadjusted					Adjusted (n = 3268)^a^			
		Estimate	SE	t value	*p* value		Estimate	SE	t value	p value
						(Intercept)	69.7	3.29	21.16	< 0.001
Bedtime at 3 years of age										
18:00–20:00	(Intercept)	70.31	0.99	70.46	< 0.001		0 (Reference)			
21:00		– 2.76	1.11	– 2.47	0.013		–2.01	1.21	– 1.66	0.097
22:00		– 3.46	1.15	– 3.01	0.003		–1.86	1.25	– 1.49	0.137
≥ 23:00		– 8.51	1.65	– 5.14	< 0.001		–5.28	1.85	– 2.85	0.004

^a^Adjusted for sex, gestational age, birth month, maternal age at delivery, maternal smoking at pregnancy confirmation, economic status, television or electronic media viewing at 3 years of age, mental developmental delay at 3 years of age.

SE, standard error.

Unadjusted analysis identified bedtime at 3 years of age and all covariates to be associated with academic performance in math. The score was 3.46 points lower for bedtime at 22:00 and 8.51 points lower for bedtime at ≥ 23:00 than for bedtime at 18:00–20:00. Multiple regression analysis adjusted for the above variables revealed that the math score was lower for children whose bedtime was at ≥ 23:00 than for children whose bedtime was at 18:00–20:00.

### Relationships among non-cognitive skills and variables at 3 years of age

Tables 4, 5 and 6 present the multiple logistic regression models used to identify the relationships between non-cognitive skills in the first grade of elementary school among the variables. Self-esteem was low in 546 (13.4%) children. Bedtime was not associated with self-esteem.

**Table 4:** Relationship between self-affirmation in the first grade of elementary school and bedtime at 3 years of age.

	Self-esteem, low (n = 546)	Self-esteem, high (n = 3541)	Unadjusted OR (95% CI)	Adjusted OR (95% CI) (n = 3036)^a^
Bedtime at 3 years of age				
18:00–20:00	58 (12.3%)	412 (87.7%)	1 (Reference)	1 (Reference)
21:00	245 (12.9%)	1651 (87.1%)	1.05 (0.78–1.44)	1.15 (0.81–1.62)
22:00	201 (13.8%)	1257 (86.2%)	1.14 (0.84–1.56)	1.15 (0.81–1.65)
≥ 23:00	42 (16.0%)	221 (84.0%)	1.35 (0.87–2.07)	1.20 (0.72–1.99)

^a^Adjusted for sex, gestational age, birth month, maternal age at delivery, maternal smoking at pregnancy confirmation, economic status, television or electronic media viewing at 3 years of age, mental developmental delay at 3 years of age.

CI, confidence interval; OR, odds ratio.

**Table 5:** Relationship between diligence in the first grade of elementary school and bedtime at 3 years of age.

	Diligence, low (n = 450)	Diligence, high (n = 3637)	Unadjusted OR (95%CI)	Adjusted OR (95%CI) (n = 3036)^a^
Bedtime at 3 years of age				
18:00–20:00	33 (7.0%)	437 (93.0%)	1 (Reference)	1 (Reference)
21:00	214 (11.3%)	1682 (88.7%)	1.68 (1.17–2.51)	1.98 (1.27–3.09)
22:00	167 (11.5%)	1291 (88.5%)	1.71 (1.18–2.57)	2.15 (1.37–3.38)
≥ 23:00	36 (13.7%)	227 (86.3%)	2.10 (1.27–3.47)	2.33 (1.29–4.20)

^a^Adjusted for sex, gestational age, birth month, maternal age at delivery, maternal smoking at pregnancy confirmation, economic status, television or electronic media viewing at 3 years of age, mental developmental delay at 3 years of age.

CI, confidence interval; OR, odds ratio.

**Table 6:** Relationship between kindness in the first grade of elementary school and bedtime at 3 years of age.

	Kindness, low (n = 433)	Kindness, high (n = 3654)	Unadjusted OR (95% CI)	Adjusted OR (95% CI) (n = 3036)^a^
Bedtime at 3 years of age				
18:00–20:00	39 (8.3%)	431 (91.7%)	1 (Reference)	1 (Reference)
21:00	187 (9.9%)	1709 (90.1%)	1.21 (0.85–1.76)	1.46 (0.96–2.21)
22:00	170 (11.7%)	1288 (88.3%)	1.46 (1.02–2.13)	1.76 (1.15–2.68)
≥ 23:00	37 (14.1%)	226 (85.9%)	1.81 (1.12–2.92)	2.15 (1.23–3.76)

^a^Adjusted for sex, gestational age, birth month, maternal age at delivery, maternal smoking at pregnancy confirmation, economic status, television or electronic media viewing at 3 years of age, mental developmental delay at 3 years of age.

CI, confidence interval; OR, odds ratio.

Diligence was low in 450 (11.0%) children. Bedtime was associated with diligence. Children who went to bed later had low diligence (21:00 vs. 18:00–20:00, OR: 1.98, 95% CI 1.27–3.09; 22:00 vs. 18:00–20:00, OR: 2.15, 95% CI 1.37–3.38; and ≥ 23:00 vs. 18:00–20:00, OR: 2.33, 95% CI 1.29–4.20).

Kindness was lesser in 433 (10.6%) children. Bedtime was associated with kindness; children who went to bed later had lesser kindness (22:00 vs. 18:00–20:00, OR: 1.76, 95% CI 1.15–2.68; and ≥ 23:00 vs. 18:00–20:00, OR: 2.15, 95% CI 1.23–3.76).

### Comparison of characteristics between included and excluded cases

Supplementary Table 1 presents a comparison of the characteristics between included and excluded cases. Sex, birth month, maternal age at delivery, and maternal smoking at pregnancy confirmation were similar between included and excluded cases. Gestational age was different between groups; the proportion of preterm infants was higher in the excluded group. Economic status was different between groups; the proportion of people on public financial assistance for daily life was higher in the excluded group.

## Discussion

This study found that bedtime at 3 years of age was associated with national language and math performance in the first grade, independent of sex, economic status, and mental developmental delay. Few studies have examined the relationship between sleep in early childhood and academic performance achievement in school and later years. Moreover, this population-based cohort study is the first to report a positive association between early bedtime and high non-cognitive skills in elementary school-aged children. These results are noteworthy as an important example of how lifestyle at preschool age may affect a child’s future cognitive and non-cognitive abilities.

Although a number of studies have examined the association between sleep and academic performance, most were cross-sectional^4^. The systematic review by Short et al. included 15 observational studies^4^ and 10 cross-sectional studies in the meta-analysis^15–24^. This analysis of 1502 schoolchildren aged 6–13 years showed that sleep duration was positively correlated with cognition^4^. Specifically, longer sleep duration improved the full intelligence quotient (IQ) and verbal IQ; however, cognitive domains such as memory, processing speed, and attention did not improve^4^. Additionally, the largest study in this meta-analysis failed to find a relationship between sleep duration and academic achievement in math or reading comprehension^15^.

In contrast to cross-sectional studies in schoolchildren, our study revealed the impact of early-childhood sleep habits on later academic performance. Bernier et al. reported no linear association between sleep duration at 2, 3, or 4 years of age and academic performance in the first grade of elementary school^12^. However, a rapid decrease in sleep duration between the ages of 2 and 4 years was associated with better scores in reading and math^12^. Kocevska et al. reported the relationship between sleep at 2 years of age and cognitive performance at 6 years of age in a population-based prospective cohort study^13^. Relative to the sleep duration at 2 years of age (11–14 h) recommended in the American Academy of Sleep Medicine guidelines^25^, 2-year-olds who slept more had lower IQ scores at 6 years of age^13^. Interestingly, 2-year-olds who slept less also tended to have lower IQ scores at 6 years of age^13^. Similarly, our study showed no linear association between sleep duration at 3 years of age and cognition during the first grade of elementary school. However, this study emphasized the importance of sleep habits in early childhood with a linear association between bedtime at 3 years of age and academic performance in the first grade.

Considering that bedtime, rather than sleep duration, was more associated with cognition, this may show the relevance of circadian rhythm. Circadian rhythm is widely associated with human health^26^. Circadian rhythm disturbance is common in patients with neurodevelopmental disorders such as autism spectrum disorder, psychiatric diseases, and dementia^26^. Circadian rhythm and sleep-wake function are essential for cell function, neural connectivity, and plasticity^27^. Conversely, neurodevelopmental disorders affect sleep-wake rhythm^27^. To increase the circadian amplitude, bright light during daytime and avoidance of light at night are important^26^. Moreover, melatonin supplementation improves sleep-wake scheduling^26,28^. Children with autism have poor nighttime melatonin secretion^29^; melatonin supplementation improves sleep habits and daytime behavior^30^. An optimum amount of sleep is crucial, and sleep restriction or deprivation leads to cognitive decline^31,32^. However, in preschool-aged children, decreased napping was associated with higher cognition, suggesting that decreased napping indicates biological brain maturation^31^. In our study, sleep duration did not correlate with future cognitive function, indicating that individuals with earlier maturation require less sleep.

Sleep habits in early childhood were also associated with future non-cognitive skills. Non-cognitive skills consist of several components such as academic motivation, diligence, responsibility and persistence, temperament, sociability, and self-esteem^8^. Non-cognitive skills are crucial for life success^8^. Heckman et al. proposed that life success including high income, reduced crime involvement, and better health was not caused by cognitive abilities, but rather non-cognitive skills^9^. Studies have also assessed the infleunce of socioeconomic status, health problems, and other environmental factors in childhood on future non-cognitive skills^33–35^. Family income, maternal education, and health problems such as difficulty in hearing and eating were associated with non-cognitive skills in elementary school^33,35^. Parental smoking also affects both cognitive and non-cognitive skills^34^. Our results are the first to suggest an association between early-childhood sleep habits and non-cognitive abilities in the later life.

The multiple regression analysis included several covariates. Covariates in this study have been reported to be associated with academic and cognitive performance. Sex and developmental milestones are widely associated with IQ at 5–8 years of age^36,37^. Very preterm children have deficits in academic achievement^38^. Birth month is also associated with academic achievement^39^. Relatively older schoolchildren in primary schools score significantly higher on academic achievement tests than do their relatively younger counterparts^39^. Parental smoking is also associated with worse developmental outcomes in children^34^. Parental economic status also affects academic performance^40–42^. Khanam et al. reported that family income had a significant positive effect on most cognitive outcomes in chidlren, but not on most non-cognitive or behavioral developmental outcomes^41^.

We performed multiple regression analysis adjusted for the above covariates to reduce confounding bias. However, there were residual confounding factors. Parental intelligence and education are important factors; however, we could not adjust for these factors because of the lack of relevant data in this study. Many studies have shown that parental education and intelligence are associated with academic performance and IQ in children^43–46^. One of these studies found that parental and child IQ associations were explained by the home environment^46^. Interestingly, another study showed that parental and child IQ associations were partly explained by the role of cardiorespiratory fitness^44^.

With the advantage of being a population-based cohort study, our results can be applied to the general population, which is a unique feature and strength of this study. However, this study also had some limitations. First, nearly half the cases were not followed through elementary school, which affects the study’s generalizability. We presumed that the main reason for exclusion was relocation. There were many cases of relocation because of Japanese customs (giving birth in their hometown and returning to the municipality where they work). Because this study was based in Amagasaki City, we could not follow-up with children who relocated to other cities. Thus, data on sleep habits and mental developmental delay at 3 years of age were not available for the excluded group. Among the comparable data, there were differences between groups in the number of weeks of pregnancy and economic status. Second, the results do not show that sleep in early childhood directly affects future academic or non-cognitive performance. Sleeping habits may have been influenced by family economic status, environmental, and genetic factors of the children, which may have affected their cognitive and non-cognitive abilities. Nevertheless, sleep habits were also independently associated with academic achievement and non-cognitive skills in multivariate analyses performed to reduce bias due to economic status and developmental delay at 3 years of age. This suggests that optimum sleep habits in early childhood may positively impact the future.

Another important limitation is measurement bias. Non-cognitive skills were assessed on the basis of self-reporting by children, which is not an objective indicator. Socioemotional and motivational skills are routinely measured using self-reports in many studies, although measurement by observation of behavior has also been attempted^47^. Nevertheless, the self-reports of first graders are still imprecise, and further validation is needed to assess their non-cognitive abilities in the future. Academic performance was originally prepared for this research project and was not conducted elsewhere. The lack of validation in other populations limits the interpretation of the results.

## Conclusion

Bedtime at 3 years of age was significantly associated with academic performance and non-cognitive skills, such as high dilligence and more kindness, in the first grade of elementary school. Although the lack of validation of measurements is a major limitation, our findings indicate that optimum sleep habits in early childhood may have a positive impact in the future of these children. Moreover, our findings may help in improving the overall lifestyle of infants and toddlers as well as prove beneficial in child health.

### Supplementary Information

Supplementary Table 1.

## Data Availability

Data are not publicly available. However, data may be obtained from the appropriate section of the Amagasaki City upon reasonable request. The corresponding author should be contacted regarding requests for data.
