# Supplementary material for: Association of early bedtime at 3 years of age with higher academic performance and better non-cognitive skills in elementary school
Source: Sci Rep. 2023 Nov 27;13:20926. doi: 10.1038/s41598-023-48280-5 (PMC10684487; doi:10.1038/s41598-023-48280-5)
Supplement: Supplementary file 1 — Supplementary Table 1. [file 41598_2023_48280_MOESM1_ESM.docx]

**Supplementary Table**

**Association of early bedtime at 3 years of age with higher academic performance and better non-cognitive skills in elementary school**

Masahiro Nishiyama, MD, PhD^a,b*^, Yuki Kyono, MD^a^, Hiroshi Yamaguchi, MD, PhD, DVM^a^, Aoi Kawamura, MD^a^, Shizuka Oikawa, MD^a^, Shoichi Tokumoto, MD, PhD^a^, Kazumi Tomioka, MD, PhD^a^, Kandai Nozu, MD, PhD^a^, Hiroaki Nagase, MD, PhD^a^

^a^ Department of Pediatrics, Kobe University Graduate School of Medicine, Kobe, Japan

^b^ Department of Neurology, Hyogo Prefectural Kobe Children’s Hospital, Hyogo, Japan

*Corresponding author:

Masahiro Nishiyama, M.D, Ph.D
Department of Pediatrics, Kobe University Graduate School of Medicine
7-5-2, Kusunoki-Cho, Chuo-Ku, Kobe, Hyogo 650-0017 Japan
Tel.: +81-78-382-6090, Fax: +81-78-382-6099
E-mail: [nishiya@med.kobe-u.ac.jp](mailto:nishiya@med.kobe-u.ac.jp)

**Supplementary Table 1** Comparison of characteristics between included and excluded cases

|  | Included children  (n=4395) | Excluded children (n=3697) | P value |
| --- | --- | --- | --- |
| Sex |  |  | 0.360 |
| Male | 2238 (50.9%) | 1921 (52.0%) |  |
| Female | 2157 (49.1%) | 1776 (48.0%) |  |
| Gestational age (weeks)^a^ |  |  | 0.005 |
| 22–32 | 34 (0.8%) | 50 (1.4%) |  |
| 33–36 | 185 (4.2%) | 189 (5.1%) |  |
| 37–43 | 4175 (95.0%) | 3452 (93.5%) |  |
| Birth month |  |  | 0.078 |
| April–September | 2264 (51.5%) | 1831 (49.5%) |  |
| October–March | 2131 (48.5%) | 1866 (50.5%) |  |
| Maternal age at delivery (years)^b^ |  |  | 0.233 |
| <20 | 60 (1.7%) | 51 (1.9%) |  |
| 20–34 | 2568 (73.0%) | 1974 (74.2%) |  |
| ≥35 | 888 (25.3%) | 632 (23.8%) |  |
| Maternal smoking at pregnancy confirmation^c^ |  |  | 0.084 |
| No | 2807 (83.7%) | 2172 (82.0%) |  |
| Yes | 546 (16.3%) | 476 (18.0%) |  |
| Economic status |  |  | 0.028 |
| No public financial assistance for daily life | 4292 (97.7%) | 3580 (96.8%) |  |
| Public financial assistance for daily life | 103 (2.3%) | 117 (3.2%) |  |

Fisher’s exact test was conducted.

^a^Data missing for <10% children.

^b^Data missing for 10–20% children.

^c^Data missing for 20–30% children.
